# Sentinel surveillance for travellers' diarrhoea in primary care

**DOI:** 10.1186/1471-2334-7-126

**Published:** 2007-11-06

**Authors:** Gemma Northey, Meirion R Evans, Tinnu S Sarvotham, Daniel R Thomas, Tony J Howard

**Affiliations:** 1 School of Medicine, Cardiff University, Temple of Peace and Health, Cathays Park, Cardiff, UK; 2 National Public Health Service for Wales, Temple of Peace and Health, Cathays Park, Cardiff, UK; 3 School of Medicine, Grove Building, University of Wales Swansea, Singleton Park, Swansea, UK

## Abstract

**Background:**

Travellers' diarrhoea is the most common health problem among international travellers and much of the burden falls on general practitioners. We assessed whether sentinel surveillance based in primary care could be used to monitor changes in the epidemiology of travellers' diarrhoea.

**Methods:**

A sentinel surveillance scheme of 30 volunteer general practices distributed throughout Wales provides weekly reports of consultations for eight infectious diseases to the national Communicable Disease Surveillance Centre. Travellers' diarrhoea was introduced as a new reportable infection in July 2002.

**Results:**

Between 1 July 2002 and 31 March 2005 there were 90 reports of travellers' diarrhoea. The mean annual consultation rate was 15.2 per 100,000 population (95% confidence interval: 12.2–18.7), with the highest rates in summer, in people aged 15–24 years, and in travellers to Southern Europe. A higher proportion of travellers than expected had visited destinations outside Europe and North America when compared to the proportion of all United Kingdom travellers visiting these destinations (38% vs. 11%; Chi^2 ^= 53.3, p < 0.0001).

**Conclusion:**

Sentinel surveillance has the potential to monitor secular trends in travellers' diarrhoea and to help characterise population groups or travel destinations associated with higher risk.

## Background

Over 60 million visits abroad are now made by United Kingdom (UK) residents each year, including 12 million visits to countries other than those in the European Union or North America [1]. Travellers can act as couriers, sentinels, and transmitters of disease [2], and can provide useful information about the presence and level of risk from infections in other countries [3-6]. Clustering of infection in travellers may alert public health authorities to a disease outbreak, or can be used to promptly warn outbound travellers of a particular hazard. Systematic collection of information about infections in travellers is therefore important. Two multinational surveillance schemes for imported infection have been established: GeoSentinel, a network of 22 sentinel travel clinics (14 in the United States and 8 in other countries) [3], and TropNetEurop based on 37 clinical sites (mostly hospital infectious diseases departments) in 14 European countries [4]. However, neither of these is particularly suited to the surveillance of travellers' diarrhoea (TD) since most cases present in primary care.

TD is the most common health problem among international travellers and has been estimated to affect 30–50% of travellers from industrialised nations who visit developing countries [7]. There are no good data on the true incidence of TD in the community or on the burden of infection presenting to general practice, and the only studies, to date, have been in individual general practices [8]. We used an established sentinel surveillance scheme based in primary care to obtain an estimate of the incidence of TD presenting to general practitioners, to monitor seasonal and secular trends, and to describe its epidemiological characteristics.

## Methods

A sentinel surveillance scheme consisting of 30 volunteer general practices distributed throughout Wales routinely reports cases of eight infectious diseases to the Communicable Disease Surveillance Centre of the National Public Health Service for Wales. It covers a total population of 215,642 registered patients (in 2003) based on the sum of the individual practice populations, representing 7.5% of the general population of Wales. The scheme was established in 1985 and provides data on chickenpox, influenza, measles, mumps, rubella, shingles, and whooping cough. In 1997, bloody diarrhoea was introduced as a reporting category and the network was used to estimate the incidence, describe the epidemiology, and investigate the aetiology of bloody diarrhoea [9]. In July 2002, travellers' diarrhoea was introduced as a new reportable infection to the scheme, replacing bloody diarrhoea. TD was defined as 'diarrhoea (3 or more loose stools in 24 hours) starting whilst abroad or within seven days of return'. Each general practice reports weekly on consultations for TD and provides, for each case, details of age group, sex, week of consultation, and countries visited.

Data were analysed by time, person, and place. We calculated rates by sex, age group, and travel destination using the combined practice populations for 2003 as the denominator and used the Poisson distribution to calculate 95% confidence intervals (CI). Travel destination was also analysed by comparing the proportion of study cases travelling to various destinations with denominator data on visits abroad by UK residents obtained from the 2003 International Passenger Survey [1].

## Results

**Figure 1:**
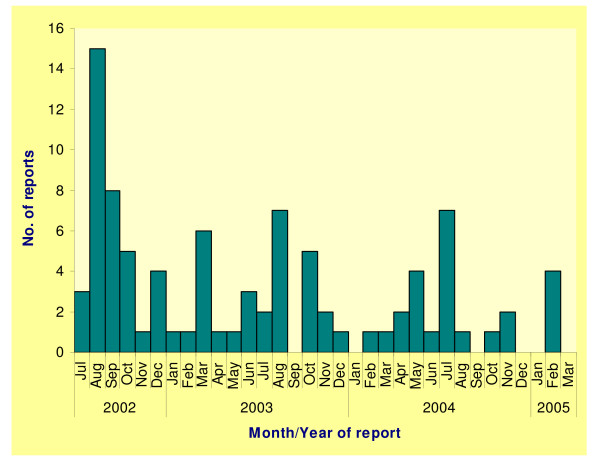
Reports of travellers' diarrhoea to the primary care sentinel scheme, Wales, Jul 2002 – Mar 2005.

A total of 90 cases of TD were reported from 17 practices during the 33 months of the study, giving a mean annual rate of 15.2 (95% CI 12.2–18.7) per 100,000 population. These comprised 36 cases from July 2002 through December 2002, 30 cases from January through December 2003, 20 cases from January through December 2004 and 4 cases from January through March 2005 (Figure 1). The highest number of consultations (24) occurred during the third quarter of 2002 at the end of the summer holiday period. There was a fall off in numbers of reports during 2003 and 2004, although high numbers continued to occur during the July–September quarter. Distinct peaks were evident in August 2002, the first full month of the sentinel study, and in August 2003 and July 2004. No practice reported more than three cases in a single week. Mean annual reporting rates for TD by practice varied between 2.9 and 96.8 per 100,000 population. High TD reporters were different from the top reporting practices for other diseases in the sentinel scheme.

There was no difference in the mean annual rate for men (16.1 per 100,000, 95%CI 11.8–21.4) and women (14.0 per 100,000, 95%CI 10.1 to 18.9) (Table 1). There was considerable variation in mean annual rates between age groups, though confidence intervals overlap. The highest rates were in the 15–24 year age group (29.1 per 100,000, 95%CI 18.0–44.5), affecting both sexes equally, and in the 45–64 year age group (20.0 per 100,000, 95%CI 13.6–28.5), with a predominance in men. Information on travel destination was available for 72 cases. Most travellers had returned from European Union countries (35/72, 49%), predominantly Spain (25/72, 35%). A higher proportion of cases reported to the sentinel scheme had visited destinations outside Europe and North America when compared to the proportion of visits to these destinations made by UK travellers generally (27/72, 38% vs. 11%; Chi^2 ^= 53.3, P < 0.0001). Most of these cases had visited either North Africa (mainly Egypt) or Asia (mainly India).

**Table 1:** Mean annual consultation rates for traveller's diarrhoea (per 100,000 practice population) by sex, age, and destination as reported by sentinel general practices in Wales, Jul 2002 – Mar 2005.

**Variable**	**No.**	**(%)**	**Consultation rate (95% CI)**
Sex			
Male	47	(52)	16.1 (11.8–21.4)
Female	42	(47)	14.0 (10.1–18.9)
Not known	1	(1)	
Age group (years)			
≤4	4	(4)	13.8 (3.8–35.3)
5–14	8	(9)	11.4 (4.9–22.4)
15–24	21	(23)	29.1 (18.0–44.5)
25–34	13	(14)	18.3 (9.8–31.3)
35–44	8	(9)	9.6 (4.2–19.0)
45–64	31	(34)	20.0 (13.6–28.5)
65+	3	(3)	2.7 (0.5–7.8)
Not known	2	(2)	
Destination			
European Union	35	(39)	5.9 (4.1–8.2)
Other Europe	10	(11)	1.7 (0.8–3.1)
North America	0	(0)	
Other areas	27	(30)	4.6 (3.0–6.6)
Not known	18	(20)	
All cases	90	(100)	15.2 (12.2–18.7)

## Discussion

The adoption of TD in our sentinel surveillance scheme was straightforward and we received regular reports from participating practices, albeit at relatively low levels. The data show a clear seasonal pattern in consultation rates with most occurring in late summer and early autumn. This corresponds with the peak in numbers of UK residents travelling aboard [1]. Reporting rates were highest in the first three months of the scheme perhaps indicating subsequent reporting fatigue, though summer peaks are still discernible each year. The highest consultation rate was in the 15–24 year age group, consistent with the high TD incidence described in this age group [7,10,11]. There was also a second peak in the 45–64 year age group maybe reflecting higher consultation rates or travel to more exotic destinations. Low rates in people aged 65 years and over are also consistent with data from other sources [10,11], and may reflect pre-existing immunity, travel to less exotic destinations, or less risk-taking behaviour whilst abroad. The range of countries visited probably reflects the most popular holiday travel destinations. Reports from people travelling to less popular destinations such as Eastern Europe, Egypt, and India may reflect higher rates of illness in these countries [7], or more serious infections, or a greater inclination to consult the doctor after return. However, the greater burden of TD will arise from visitors to countries such as Spain which is the most popular destination for UK residents, accounting for 30% of holidays abroad [1].

The annual consultation rate of 15.2 per 100,000 population for TD is considerably less than estimates derived from a national health survey carried out in Wales in 1998 (80 per 100,000) [11]. This probably reflects under-reporting in the sentinel scheme, but could also be due to change in consultation habits over time, or over-reporting in the national survey due to the use of a loose case definition and reliance on self-reporting by patients. Wide variation in practice reporting rates in our study again suggests an under-reporting problem rather than true differences in TD incidence in different parts of Wales. Although sentinel surveillance schemes are generally considered to provide better data than statutory notification systems [12], very few studies have tried to assess completeness of reporting. One study of sentinel surveillance for non-communicable diseases in primary care found reporting to be poorer for less clearly defined diseases [13]. Another study found that reporting is very dependent on the motivation of participating general practitioners and tends to be better for uncommon or serious conditions [14].

Several attempts have been made to estimate or monitor the incidence of TD. Most of these are based on ad hoc studies in returning travellers or cohort studies of special groups such as Peace Corps volunteers [15] or military personnel [16]. These approaches are of limited value in tracking changes in epidemiology or in identifying outbreaks, both of which require ongoing surveillance. In the UK, private tour operators have developed a crude surveillance tool based on consumer satisfaction questionnaires completed by returning travellers [17]. It has proved useful in identifying problems in specific countries or holiday resorts, and occasionally even at individual hotels, but is limited to adult package holiday travellers. In Japan, an electronic network for monitoring TD based on two major airport quarantine stations and three infectious disease hospitals has proved its value in identifying clusters of infection among travellers, including a salmonella outbreak [18]. Both these schemes rely on establishing special arrangements for surveillance. Many areas, however, already operate sentinel surveillance schemes, yet their potential use for the surveillance of TD has not been exploited.

This sentinel scheme for TD is still in its infancy. It provides data on TD, particularly destination of travel, that are not currently available from the two main surveillance systems in Wales: clinical notifications of food poisoning and laboratory reporting of gastrointestinal pathogens. As more data accumulate over time, the ability of the system to track secular trends and identify changes in patterns will improve. Reporting rates can be affected by changes in consultation patterns, but it should be possible to distinguish the two by comparison with reporting rates for other infections in the sentinel scheme and with routinely available health service data on consultation rates. Denominator data on the destination of travellers abroad is routinely collected in the United Kingdom by the annual International Passenger Survey [1], and this provides the potential to analyse TD rates for specific countries. In the future, with the development of computer systems in primary care, it may be possible to electronically extract data that is suitable for use in infectious disease surveillance.

## Conclusion

This study shows the feasibility of incorporating TD in a primary care sentinel scheme. Although it suffers from under-reporting, it nevertheless has the potential to monitor secular trends, and to help characterise population groups or travel destinations associated with higher risk.

## Competing interests

The author(s) declare that they have no competing interests.

## Authors' contributions

MRE conceived and designed the study and drafted the manuscript. GN and TSS coordinated data collection and analysis, and helped draft the manuscript. DRhT and TJH helped design the study and revise the draft manuscript. All authors read and approved the final manuscript.

## Pre-publication history

The pre-publication history for this paper can be accessed here:


